# Glucose-Responsive Boronic Acid Hydrogel Thin Films Obtained via Initiated Chemical Vapor Deposition

**DOI:** 10.1021/acs.biomac.2c00762

**Published:** 2022-09-02

**Authors:** Katrin Unger, Anna Maria Coclite

**Affiliations:** Institute of Solid State Physics, NAWI Graz, Graz University of Technology, 8010 Graz, Austria

## Abstract

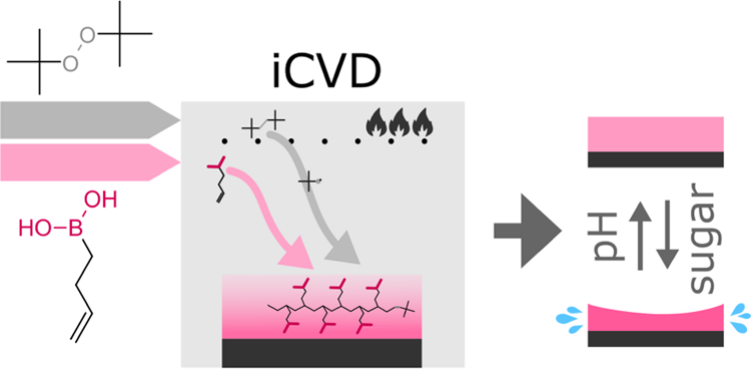

Glucose-responsive materials are of great importance in the field of monitoring the physiological glucose level or smart insulin management. This study presents the first vacuum-based deposition of a glucose-responsive hydrogel thin film. The successful vacuum-based synthesis of a glucose-responsive hydrogel may open the door to a vast variety of new applications, where, for example, the hydrogel thin film is applied on new possible substrates. In addition, vacuum-deposited films are free of leachables (e.g., plasticizers and residual solvents). Therefore, they are, in principle, safe for in-body applications. A hydrogel made of but-3-enylboronic acid units, a boronic acid compound, was synthesized via initiated chemical vapor deposition. The thin film was characterized in terms of chemical composition, surface morphology, and swelling response toward pH and sucrose, a glucose–fructose compound. The film was stable in aqueous solutions, consisting of polymerized boronic acid and the initiator unit, and had an undulating texture appearance (rms 2.1 nm). The hydrogel was in its shrunken state at pH 4–7 and swelled by increasing the pH to 9. The p*K*_a_ was 8.2 ± 0.2. The response to glucose was observed at pH 10 and resulted in thickness shrinking.

## Introduction

The possible biological applications for smart glucose-responsive materials span from smart drug encapsulation, especially for delivering insulin for diabetes patients, over biological sensors for tracking the glucose amount in blood or sweat to glucose-triggered actuators such as opening pores of membranes.^1−3^ Three typical candidates that provide selective glucose responsiveness are glucose oxidase, an enzyme that catalyzes the oxidation of glucose to gluconolactone and hydrogen peroxide, lectin-based systems, a protein that regulates hormones on the surface of cells, and boronic acid compounds, which consist of a boron atom bonded to two OH groups and a residual group.^1^

So far, the synthesis of glucose-responsive materials was performed via solution-based deposition methods.^4−6^ An example is based on the boronate esterification reaction of the diol groups of poly(vinyl alcohol) with the boronic acid groups of 2-acrylamidophenylboronic acid in aqueous conditions.^4^ Other examples of interesting boronic acid-based hydrogels obtained in solution are the biosynthetic hybrid ones, formed by mixing aqueous solutions of biohydrogels (e.g., mucin^5^ or alginate^7^) with synthetic polymers carrying boronic acid groups. These methods are attractive fabrication processes, as they can be performed under ambient conditions and are easily scalable. Extending the delivering routine of glucose-responsive materials also to vacuum-based techniques would provide a new tool with the benefits of a leachable (e.g., solvent, plasticizer)-free, conformal, and substrate-independent process for the deposition of such materials as thin films.

**Scheme 1:**
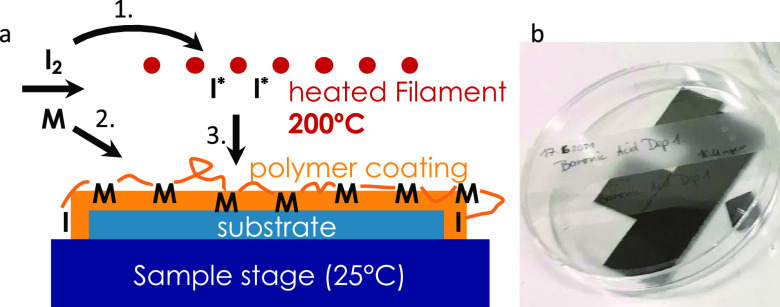
(a) Representation of the iCVD Polymerization Process During step 1, the initiator (I_2_) and monomer (M) vapors enter the reactor chamber. In step 2, the monomer absorbs on the surface of the substrate. In step 3, the heated filament (kept at 200 °C) decomposes the initiator into radicals, which initiate polymerization on the surface. (b) Photograph of some hydrogel thin films deposited on silicon wafers.

Within this study, the first glucose-responsive material delivered via a vacuum-based technique, named initiated chemical vapor deposition (iCVD), will be presented. iCVD was invented by the group of Gleason at MIT in 2006.^8,9^ A schematic of this process is shown in Scheme 1a. The initiator (indicated in the scheme as I_2_) and monomer (indicated in the scheme as M) species enter the chamber as vapors. The labile bond in the initiator (e.g., O–O in di-*tert*-butylperoxid) decomposes at a relatively hot filament (200 °C). At the surface, the radical and the adsorbed monomer species react via a free radical chain polymerization reaction.^10,11^ The polymerization takes place from the surface and upwards, forming a thin film with high uniformity and conformality. A photograph of glucose-responsive thin films deposited on silicon wafers is shown in Scheme 1b.

With iCVD, smart hydrogels responding to temperature, humidity, light, and pH were synthesized.^12−19^ The great advantages of iCVD, compared to other vacuum-based deposition methods for organic compounds such as plasma-enhanced CVD or photoinduced CVD, are the mild process conditions resulting in a substantial retention of functional groups and a great variety of applicable substrates, such as tissue paper, pharmaceutics, optical microcavity probes, or even ionic liquids,^20−25^ while on the contrary, for the monomer species, certain requirements need to be fulfilled. They need to be vaporizable and contain a vinyl (C=C) bond, which will form the polymer backbone during the synthesis. Glucose oxidase and lectin-based systems are massive molecules that tend to decompose before they vaporize. For that reason, the monomer used in this study is but-3-enylboronic acid, and is, as the name already suggests, a boronic acid compound. Boronic acid hydrogels were already successfully implemented in various applications such as glucose-triggered release of insulin, electrochemical or optical glucose sensors, or scaffolds for cell cultures.^26^

This paper wants to be a proof-of-concept study on the deposition of glucose-responsive hydrogels by iCVD, addressing whether a boronic acid-based hydrogel can be synthesized by this method and whether it shows the expected glucose responsiveness.

## Materials and Methods

### Boronic Acid Hydrogel Synthesis

The chemical compounds but-3-enylboronic acid (BA, Merck, Germany, catalog number 687650) and di-*tert*-butylperoxid (TBPO, Merck, Germany, catalog number 168521) were purchased and used without any further purification. Thin-film hydrogels were synthesized via initiated chemical vapor deposition (iCVD) in a custom-built reactor, as described elsewhere.^27^ The monomer powder BA was heated to 85 °C and was fed into the reactor with a flow rate of 0.23 sccm. The initiator, TBPO, was kept at room temperature and entered with a flow rate of 1 sccm. The substrate temperature and the filament temperature were set to room temperature and 200 °C, respectively. The deposition was carried out at a working pressure of 500 mTorr for 180 min. After the deposition, the sample was rinsed with demineralized water to remove unreacted monomers or loosely bound oligomers, ending up with a film thickness of 56 nm (measured with spectroscopic ellipsometry). A photograph of the thin films deposited on silicon wafers is reported in Scheme 1b.

### Characterization Methods

Via Fourier transform infrared (FTIR) spectroscopy (Bruker IFS 66v/S), the polymerization as well as the retention of functional groups were investigated. Spectra were recorded in the transmission mode in the region of 400–4000 cm^–1^ with a resolution of 4 cm^–1^ and were baseline-corrected. When measuring the monomer spectrum, the BA powder was clamped between two Si wafers, and as a reference, two empty wafers with the same spacing were used. When measuring the synthesized thin film, a plane wafer was used as a reference. The spectra were not normalized. Besides literature research, KnowItAll software was used to identify the peaks.

An atomic force microscope (AFM, Nanosurf easyScan 2, Liestal, Switzerland) was utilized to investigate the morphology and surface roughness of the polymerized layer before and after the sample was immersed in different pH and glucose media. A cantilever (Tap190Al-G, BudgetSensors, Bulgaria), operating in the tapping mode, scanned an area of 6 × 6 μm with a resolution of 1024 × 1024, a speed of 1 s/line, a P/I/D parameter of 5000/700/0, a vibration amplitude of 100 mV, and setpoints of 20 and 40% for unrinsed and rinsed samples, respectively. Data handling and statistical evaluation were performed by Gwyddion 2.56 software, an open source analysis software for height fields.^28^

By spectroscopic ellipsometry (J. A. Woollam M-2000, Lincoln, NE), the evolution of thin film thickness in different pH and glucose media was measured. With a tightly sealed liquid cell (J. A. Woollam, Lincoln, NE), the sample was exposed to different solutions, while in situ spectra were measured each 4 s at an angle of 75° in the spectral region from 370 to 1000 nm (a schematic of the setup is shown in Figure 2e). The solution within the cell (cell volume of about 4 mL) was exchanged by injecting 10 mL of a new solution with a different pH value or sucrose (10 mg/mL, Sigma-Aldrich, catalog number S0389) content into the inlet of the cell. The program CompleteEASE was used to fit the data. The sample in the dry state (measured in air) was fitted with a three-layer model consisting of a silicon substrate, a native silicon dioxide (2 nm), and a Cauchy layer, representing the hydrogel, while measuring in buffer solutions, the fitting wavelength region was cropped to 450–900 nm. The Cauchy layer was replaced by an effective material approximation model, which averaged the defined optical properties of two different materials weighted by the ratio of the materials within the layer. One material optical property was the parameters obtained for the hydrogel in the dry state, and the other material was water.

The buffer solutions were prepared as follows: for pH 4 and 5, a citric acid buffer of citric acid (0.1 M) and Na_2_HPO_4_ (0.2 M); for pH 6 and 7, a phosphate buffer (0.1 M) of Na_2_HPO_4_ and Na_2_H_2_PO_4_ was used; for pH 8 and 9, a Tris buffer of a Tris base (0.1 M) and HCl was used; and for pH 10, a standard buffer solution for pH calibration was used.

## Results and Discussion

### Chemical Characterization

**Figure 1:**
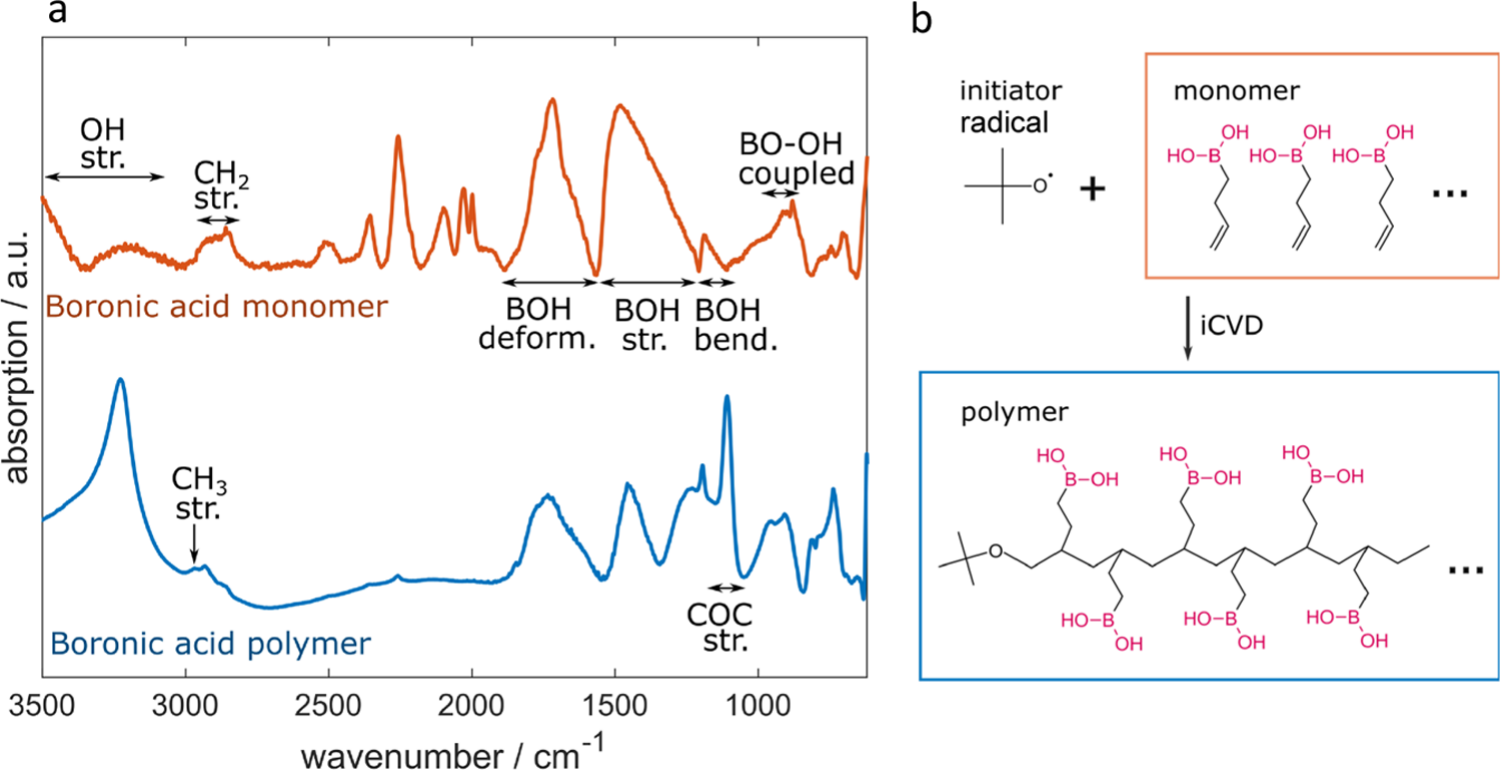
(a) FTIR spectra of the boronic acid monomer and the boronic acid polymer. (b) Free radical chain polymerization reaction.

To verify the polymer synthesis as well as the retention of the functional boronic acid groups during the iCVD process, FTIR was utilized. The spectra of the boronic acid monomer as well as of the polymer are depicted in Figure 1a. The spectrum of the monomer includes the broad O–H stretching vibration band around 3300 cm^–1^, the stretching of C–H in H–C=C at 3040–3010 cm^–1^, and the antisymmetric and symmetric stretching bands of sp^3^ C–H at 2920 and 2860 cm^–1^, respectively. Further, broad characteristic B–O–H bands caused by deformation, stretching, and bending can be found at 1730, 1450, and 1185 cm^–1^, respectively.^29−31^ Then, a double peak at 900 and 880 cm^–1^ caused by coupled B–O stretching plus O–H in-plane bending mode and a symmetric in-plane O–H bending is present. These two modes in the region of 900–1000 cm^–1^ are as well characteristic of boronic acid and differ from other alcohol derivates. Indeed, Smith et al. showed via simulations that the B–O stretch/O–H in-plane bending mode does not have an analogue outside boronic acid and can therefore show decisive characteristic features.^32^ The last peak series between 780 and 695 cm^–1^ was attributed to the surface curvature of O–H.^31^ Further, several peaks in the region of 2525 and 1980 cm^–1^ are visible. These peaks are present in other spectra of boric acid or boronic acid compounds as well.^31^ The vinyl peak (C=C) at 1640 cm^–1^, which is typically used to verify polymerization, is not visible neither in the monomer nor in the polymer spectrum because of the pronounced B–O–H deformation band.

After the polymerization, schematized in Figure 1b, the characteristic B–O–H deformation, stretching, bending, and coupled B–O and O–H bands are still present, demonstrating the retention of functional groups. The peaks between 2525 and 1980 cm^–1^ are damped or vanished after the polymerization, which was as well observed in the literature.^33^

In the C–H stretching region, a peak at 2968 cm^–1^ is present. This may be attributed to the C–H stretching in methyl groups.^34^ Since no methyl groups are present in the monomer structure, it can be assumed that their presence in the polymer chain comes from the initiator. Additionally, a band in the footprint region at 1108 cm^–1^ can be assigned to the C–O–C stretching vibration band, which is the interlink between the initiator and the polymer backbone.^29^ This peak should have an intensity between medium and strong according to the FTIR analysis software KnowItAll. The peak intensity is the highest compared to all other peaks in the footprint region (the B–O–H stretching also has strong intensity). This indicates a large percentage of chain ends and therefore shorter chains. Longer polymer chains may be achieved by optimizing the deposition parameters.

### pH and Glucose Response

**Figure 2:**
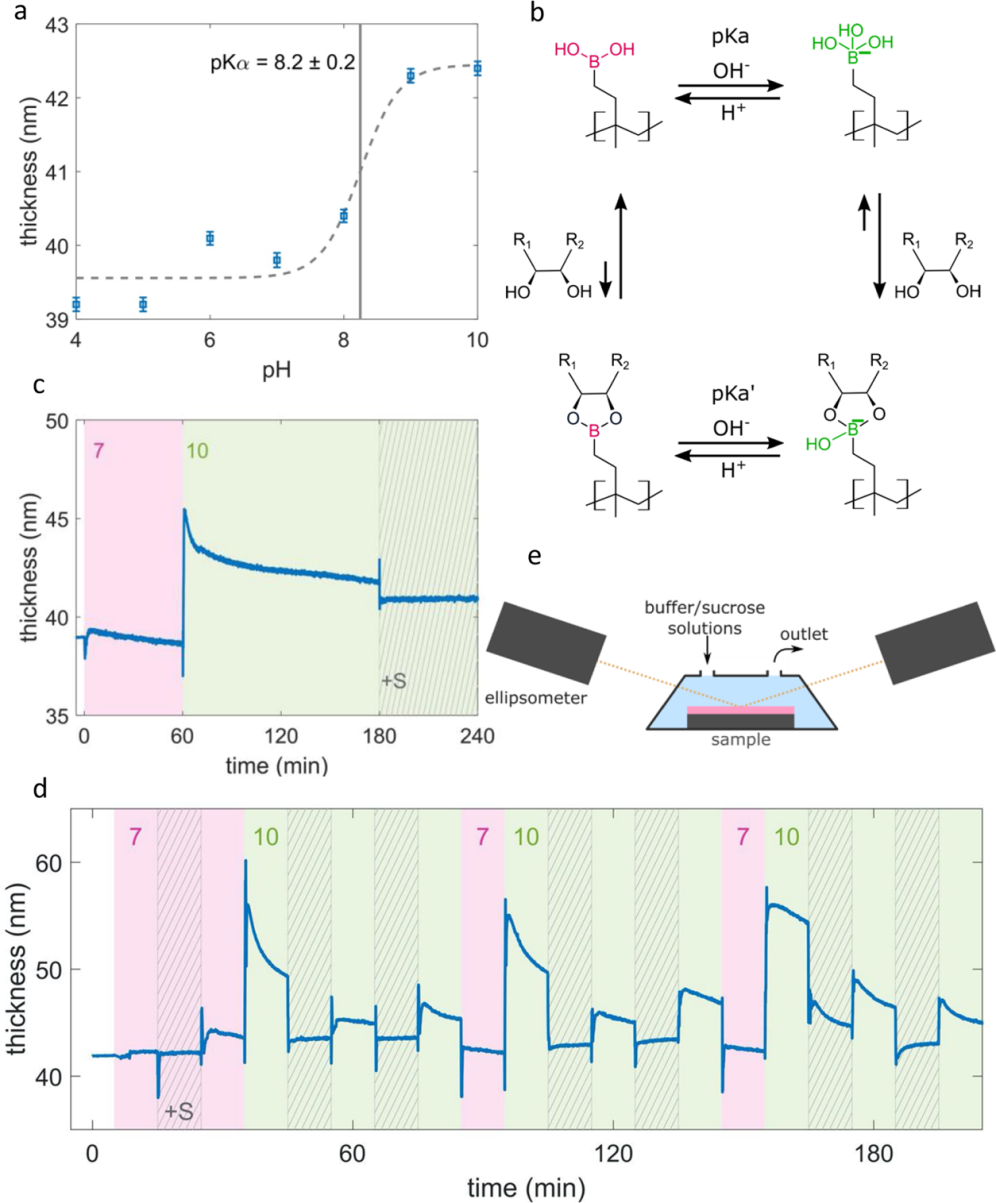
(a) Hydrogel thickness in the pH domain. Below p*K*α, a shrunken state is present, while above p*K*α, a swollen state is present. The fitted p*K*α value is 8.2 ± 0.2. (b) Reversible binding schematic between the boronic acid hydrogel and diols as present in glucose in the pH domain.^47^ (c) In situ hydrogel swelling when immersed for 1 h in pH 7, for 2 h in pH 10, and finally in pH 10 plus 10 mg/mL sucrose. (d) In situ hydrogel swelling when immersed in different buffers (pH 7 and 10) without or with sucrose. (e) Schematic of the immersion of the hydrogel in several buffer/sucrose solutions at different pH values for the in situ ellipsometric analysis.

The hydrogel pH response, in terms of thickness variation upon exposure to solutions at different pH values, was investigated by spectroscopic ellipsometry. The thickness of the sample immersed for 10 min in different buffer solutions is plotted versus the pH value in Figure 2a. At pH 4–7, the boronic acid polymer is in a shrunken state, while above pH 7, the polymer starts to swell and reaches a plateau at pH 9. The p*K*α value was estimated at 8.2 ± 0.2. Generally, boronic acids are classified as weak organic Lewis acids. In acidic conditions (pH < p*K*α), the neutral form is favored (see Figure 2b, top-left) with a vacant π-orbital.^35^ The sp^2^ hybridized boron assembles together with the two oxygen atoms and the one residual partner in a trigonal planar configuration. In more basic conditions (pH > p*K*α), the empty π-orbital is filled by a OH ion complexation to a hydroxyboronate anion (see Figure 2b, top-right). In the literature, it is shown that these two states impact the properties of the materials, such as electronic properties (from an electron-accepting to an electron-donating compound),^35^ swelling properties,^36^ or optical properties.^37−39^ By Kim, Mujumdar, and Siegel, the swelling change of phenylboronic acid from a shrunken into a swollen formation in acidic and basic conditions, respectively, was explained by, first, the increase of the hydrophilicity of boronic acid units due to a polarity change in the ionized form and, second, the water influx by osmotic pressure caused by the counterions of BO^–^.^40^

The p*K*α of different boronic acid compounds varies from 4 to 10. While the unmodified phenylboronic acid exhibits a p*K*α of 8.5,^40^ derivatives of phenylboronic acid have different p*K*α when the aromatic ring was modified with electron-withdrawing or -donating structures.^35^ Further, a modification of diol (−OH)-containing structures leads to a variation of p*K*α too.^41^

In Figure 2c, it is shown that the thickness of the hydrogel was immersed sequentially for 1 h in a buffer solution of pH 7, for 2 h in a buffer solution of pH 10, and finally for 1 h in a buffer solution of pH 10 mixed with 10 mg/mL sucrose. Such high sucrose concentration was chosen for this initial study to see a strong response of the hydrogel, as it is expected that more sucrose can react with the hydrogel. While in the dry state, the boronic acid polymer had a thickness of (38.97 ± 0.03) nm, and when immersed in a buffer solution of pH 7, a slight thickness change to a thickness of (39.32 ± 0.06) nm followed by a slight thickness decrease ending at (38.6 ± 0.1) nm can be observed. At pH 10, a rapid (<30 s) increase of thickness to (45.3 ± 0.1) nm is followed again by a thickness decrease to (41.78 ± 0.08) nm. The change from pH 7 to pH 10 from a collapsed into a swollen hydrogel is consistent with the plot shown in Figure 2a, and it can be explained considering that pH 7 is below the estimated p*K*α of the hydrogel, while pH 10 is above it. Kinetic effects are also evident from the in situ measurements in Figure 2c. At pH 7 as well as at pH 10, first, the polymer expands within minutes, and then, it retracts again way slower in time domains of hours. Kinetic studies of the humidity-responsive polyelectrolyte hydrogel multilayer were investigated by Secrist and Nolte via in situ reflectivity.^42^ They showed that the structural response happened in two time domains: an initial swelling response in the order of seconds to minutes when water enters the film and a longer time scale structural relaxation in the order of hours to days. For the results presented in this study, the combination of fast swelling due to the strong water affinity of the hydroxyboronate anion and a slow relaxation due to the rearrangement of polymer compounds might as well fit as a hypothetical explanation of the thickness evolution within one buffer solution.

Afterward, when the sample is immersed in a buffer solution of pH 10 mixed with sucrose, the thickness decreased rapidly to a constant value of (40.8 ± 0.1) nm. A schematic of the chemical reaction of the hydroxyboronate anion toward diols (such as present in saccharides) is illustrated in Figure 2 on the right side. Typically, boronic acid hydrogels swell in the presence of glucose due to an increase in the fraction of hydroxyboronate anions, which are more hydrophilic.^26^ On the contrary, Alexeev et al. proposed that glucose can simultaneously bind with two boronate units within the hydrogel, acting as a cross-linker that contracts the hydrogel matrix together and results in deswelling,^43^ as reported by other groups as well.^44,45^ Further, they showed that the hydrogel contracts up to a certain glucose molar concentration, suggesting a 1:2 binding of glucose and boronic acid units. Thereon, with increasing glucose concentration, the swelling of the hydrogel was observed, assuming a 1:1 binding ratio.^46^ In this study, deswelling was observed when the sample is immersed in pH 10 and a sucrose concentration of 10 mL/mg. Sucrose provides as well several possible diol–boronic acid reaction sites, giving a chance to interlink up to 4 boronic acid units and create a densely cross-linked structure, which would explain the hydrogel contraction of the hydrogel upon sucrose addition.

The hydrogel’s behavior to cyclic immersion in pH 7 and 10 buffer solutions without and with sucrose is plotted in Figure 2d. The solutions were changed every 10 min. The hydrogel thickness immersed in a buffer solution of pH 7 is (42.28 ± 0.07) nm. If sucrose (10 mg/ml) is added, marginal shrinking is observable (42.11 ± 0.04) nm. This might have the same reason why the hydrogel shrinks at pH 10 when sucrose is added. By incorporation of sucrose, the cross-linker density is increased and the meshes contract. While at pH 7, the unbonded boronic acid unit is favored, and it can be expected (expressed by the arrows in Figure 2b on the left)^35^ that only a few sucrose units are incorporated. An interesting behavior can be witnessed when the buffer solution is again changed to pH 7 but without sucrose. The hydrogel swells to (44.24 ± 0.05) nm and relaxes within 10 min to (43.61 ± 0.05) nm. A hypothesis might lie in the osmotic pressure acting on the polymer matrix caused by the introduced sucrose gradient. The meshes need to be opened to release the incorporated sucrose and can afterward relax again.

When the sample is afterward immersed in a pH 10 buffer solution, the film expands quickly to a thickness of (54.99 ± 0.06) nm and shrinks within 10 min to a thickness of (42.9 ± 0.1) nm, similar to what is already observed in Figure 2c. Exposing the hydrogel cyclically to solutions with and without sucrose at pH 10 induces reproducible shrinking when the sample is exposed to sucrose but a reduced response when the sucrose-free buffer is inserted compared to the first cycle. Apparently, the hydrogel’s history of previous exposures influences the following response behavior. Above p*K*α, the hydroxyboronate anion prefers the reaction with the diol, as indicated in Figure 2b with the arrows.^35^ It is likely that sucrose cannot be removed from the hydrogel when the buffer solution changes from pH 10 with sucrose to pH 10 without sucrose. Therefore, the hydrogel response is diminished after the first cycle. When immersing the hydrogel again to pH 7 (Figure 2d at 90 min), boronic acid (<p*K*α) is preferentially in the neutral state, which causes a release of all sucrose that was previously bonded to the hydroxyboronate anion. Repeating the cyclic exposure at pH 10 without and with sucrose after the sample was immersed in pH 7 leads to the same result.

### Morphological Characterization

**Figure 3:**
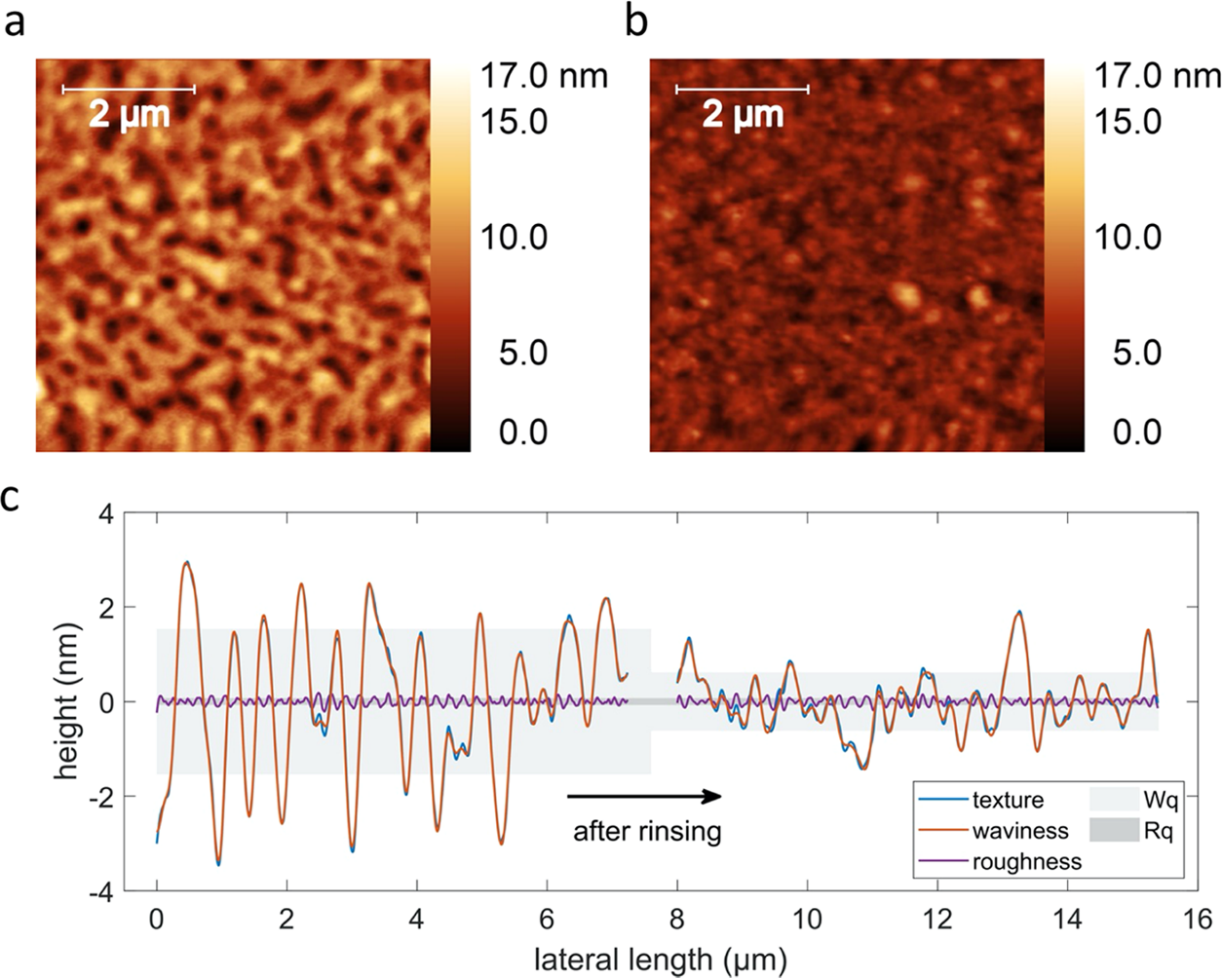
Topography images of the boronic acid hydrogel deposited via iCVD before (a) and after (b) immersion in pH buffer solutions and glucose solutions, after the measurements of Figure 2d. (c) One-dimensional (1D) height profile of both samples. The texture is the actual measurement, the waviness represents the surface topographical features in the micrometer to nanometer scale, and the roughness represents the features in the picometer scale. The gray and dark gray areas represent the root mean square of the waviness and the roughness, respectively.

The surface morphology before and after immersion in buffer solutions and glucose solutions (precisely, after the experiment performed in Figure 2d) was investigated by AFM, and it is shown in Figure 3. Before immersion, the surface appears like an undulated texture with an overall roughness of σ_rms_ = (2.1 ± 0.1) nm. After the immersion, the sample still has an undulated appearance with a lower σ_rms_ of (1.1 ± 0.1) nm.

A one-dimensional texture profile was extracted from both images and was split into waviness (low-frequency components) and roughness (high-frequency components), as plotted in Figure 3c. Before rinsing the sample, the extracted waviness is *W*_q_ = (1.5 ± 0.1) nm and the roughness is *R*_q_ = (81 ± 12) pm, which is below the height resolution of the used AFM, and the spatial wavelength of the profile (corresponding to characteristic distances between features) is λ_q_ = (140 ± 30) nm, while after immersion, *W*_q_ decreases to (0.60 ± 0.03) nm and *R*_q_ = (76 ± 7) pm and the wavelength λ_q_ = (160 ± 10) nm. This indicates that first, there is only one detectable height scale domain on the surface (in the limits of the used AFM equipment), second, the surface gets smoother after immersing the sample, and last, the spatial wavelength and therefore the characteristic lateral length between valleys and hills remain constant.

The first study and interpretation of the iCVD polymer surface morphology were done by Lau in the group of Gleason. The surface texture was attributed to the competition between the propagation rate and the nucleation rate.^48^ Lately, it was as well proposed by Perrotta et al. that the substrate and the monomer surface tension affect the topographical formation. It was shown that at elevated substrate temperatures, fluorinated poly(1*H*,1*H*,2*H*,2*H*-perfluorodecyl acrylate) tends to unwet the sample surface and can grow via islands.^49^ Besides the substrate temperature, other process parameters that impact the surface topography are the filament temperature and the saturation ratio.^50−52^ By varying these, surface morphologies of different polymers ranging from ultra-smooth over undulating to rod-like or crystal structures were achieved. As an example, Sevgili and Karaman showed a morphology change from a smooth (about 1 nm) to an undulating surface (up to 14 nm) by varying the substrate temperature.^53^ The undulating texture of their polymers, similar to the presented boronic acid surface, was explained by the domination of film growth by the initiator (more nucleation sites). The strong C–O–C peak in the FTIR of the boronic acid hydrogel presented in the FTIR results in Figure 1a indicates a large fraction of polymer starting units, which strengthens the idea of a large percentage of nucleation sites during the iCVD process. In addition, such morphology does not seem to be a characteristic of boronic acid hydrogel thin films, as in the literature, smooth films also are reported from drop-casting of polymers obtained by solution synthesis.^54^

## Conclusions

In this study, for the first time, a vacuum-based deposition routine, named initiated chemical vapor deposition (iCVD), is used to polymerize a glucose-responsive compound of a boronic acid hydrogel. This prosperous method paves new possibilities for numerous applications, including smart drug encapsulation, glucose-triggered actuators, and sensors, where the substrates cannot be coated under conventional solution-based techniques.

The polymerization of the monomer but-3-enylboronic acid is proven by FTIR, which indicates a great retention of functional units and a notable percentage of chain terminations. The surface morphology exhibits an undulating appearance (σ_rms_ = 2.1 nm), which is assigned to a prominent number of nucleation centers, which fits the high amount of chain ends.

The swelling versus the pH reveals a rising sigmoidal-shaped curve with a determined p*K*α of 8.2 ± 0.2. The change from a shrunken (at pH 4 to pH 7) to a swollen hydrogel (>pH 9) is explained by the increase of hydrophilicity and the water influx by osmotic pressure caused by the counterions of BO^–^.

The in situ swelling response toward sequentially changing pH from 7 to 10 and sucrose from 0 to 10 mg/mL demonstrates a minor response when, at pH 7, sucrose is added, while in the swollen state, at pH 10, the hydrogel shrinks more when sucrose is added. This may be explained by the multiple active bonding sites of sucrose that can interlink polymer chains and contract the meshes. Further studies with different sucrose concentrations are planned.

At pH 10, the hydroxyboronate anion favorably bonds to sucrose, which can be removed by immersing the sample in pH 7 again.
